# The validity of pediatric cancer diagnoses in a population-based general cancer registry in Ontario, Canada

**DOI:** 10.1186/s12885-016-2931-8

**Published:** 2016-11-14

**Authors:** Sumit Gupta, Jason D. Pole

**Affiliations:** 1 Division of Haematology/Oncology and Program in Child Health Evaluative Sciences, The Hospital for Sick Children, 555 University Avenue, Toronto, M5G 1X8 Canada; 2 Department of Paediatrics, Faculty of Medicine, and Institute for Health Policy, Management, and Evaluation, University of Toronto, 155 College Street, Suite 425, Toronto, M5T 3M6 Canada; 3 Cancer Research Program, Institute for Clinical Evaluative Sciences, 2075 Bayview Avenue, G1 06, Toronto, M4N 3M5 Canada; 4 Pediatric Oncology Group of Ontario, 480 University Avenue, Suite 1014, Toronto, M5G 1V2 Canada

**Keywords:** Cancer, Child, Health services research, Registry, Validation

## Abstract

**Background:**

Data from population-based cancer registries are increasingly used to conduct research and guide policy. However, few validation studies of cancer registry data have been conducted, particularly among children. We therefore aimed to determine the validity of pediatric diagnostic data in the Ontario Cancer Registry (OCR).

**Methods:**

All children diagnosed with any malignancy between 2000–2011 in Ontario, Canada were identified through the Pediatric Oncology Group of Ontario Networked Information System (POGONIS), a pediatric cancer registry actively maintained by trained data managers, and linked to the OCR. International Classification of Diseases for Oncology codes for each patient were taken from the OCR and converted to International Classification of Childhood Cancer (ICCC-3) diagnostic groups and subgroups using published algorithms. OCR-based ICCC-3 groupings were then validated by comparing them to gold standard diagnostic information from POGONIS.

**Results:**

A total of 4448 patients met inclusion criteria; 4073 (91.6 %) were successfully linked to the OCR. Diagnostic accuracy was excellent for many childhood solid tumors. For example, the OCR correctly identified all cases of retinoblastoma [kappa = 1.00, 95^th^ confidence interval (CI) 1.00–1.00] and nearly all cases of neuroblastoma (kappa = 0.97, 95^th^ CI 0.96–0.99). Hematologic and central nervous system (CNS) cancers, the most common childhood malignancies, were however often misclassified with inferior kappas (acute lymphoblastic leukemia – 0.77, 95^th^ CI 0.75–0.80; Burkitt lymphoma – 0.02, 95^th^ CI 0.02–0.07).

**Conclusions:**

Misclassification of common pediatric hematologic and CNS cancers was significant and may lead to inaccurate incidence and survival estimates using cancer registry data. Validation studies of pediatric data in other registries are necessary to identify practices and procedures leading to the highest quality information.

**Electronic supplementary material:**

The online version of this article (doi:10.1186/s12885-016-2931-8) contains supplementary material, which is available to authorized users.

## Background

Population-based cancer registries have proven an invaluable resource for both cancer researchers and policymakers [1]. Cancer registry data have been used to derive population-based estimates of outcome and to track changes in incidence in both adults and children [2–4]. In addition, when linked to other health administrative data, they have also provided a rich source with which to conduct comparative effectiveness research and guide government policy [1, 3].

Cancer registries are often considered gold standards against which other data sources are validated [5, 6]. Despite their impact upon both care and policy, very few studies have examined the validity of cancer registries themselves. Most determinations of validity have relied on indirect measures such as the percentage of cases that are microscopically verified or the proportion of cases identified only by death certificates [7]. Studies involving re-abstractions or comparisons to the source medical records are less common [8, 9]. While several studies have reported favorably when estimating the completeness of capture of childhood cancer cases in cancer registries, the ability of general cancer registries to accurately describe incident cancer cases may differ between adult and pediatric malignancies [10, 11]. Unlike adult malignancies, pediatric diagnoses are more heavily dependent on histology and not site; they also represent a heterogeneous group of malignancies that make up only 1 % of the total cancer burden [12]. Algorithms and personnel employed by registries may therefore be heavily influenced by adult oncology, and consequently fail to validly capture childhood cases. This may be of particular concern in malignancies that are unique to childhood.

Our objective was therefore to validate the diagnosis data contained in a provincial cancer registry covering all ages for a population-based cohort of pediatric oncology patients. We were able to achieve this objective by taking advantage of the unique data sources available in Ontario Canada, which include two population-based cancer registries: one general passive registry and one active pediatric-specific registry. While the completeness of these two registries has previously been compared, no study has compared specific data elements common to both [11]. Diagnosis data in the general cancer registry were therefore compared to that in the pediatric registry.

## Methods

### Data sources

The Pediatric Oncology Group of Ontario Networked Information System (POGONIS) is a population-based registry capturing data on all cases of Ontario pediatric cancer diagnosed at pediatric oncology centers. Pediatric oncology care in Ontario is delivered through five tertiary centers and their associated satellite centers. POGONIS collects data through an active process; trained data managers at each of the five tertiary centers prospectively and actively abstract demographic, disease, treatment and outcome data for all new cancer cases. Data managers routinely attend tumor boards and other medical rounds to ensure completeness and validity of the data, including diagnosis. Senior POGONIS administrators also review these data centrally to assess accuracy; data managers are routinely contacted for clarification of certain data elements, and can be asked to return to the patient chart if necessary. Data managers in turn contact treating clinicians if necessary. Previous work has shown that POGONIS identifies greater than 96 % of Ontario children with cancer aged 0–14 years [11]. Adolescents treated at adult institutions are not identifiable through POGONIS, leading to lower capture rates within POGONIS of patients aged 15–18 years.

Covering a population of approximately 13 million, the Ontario Cancer Registry (OCR) is a population-based tumor registry which relies on the passive receipt of reports from four sources: pathology reports with a diagnosis of cancer from all pathology labs across the province, hospital discharge records containing a diagnosis of cancer from all hospitals across Ontario, electronic health records from specific treatment centers, and any electronic death record with cancer as one of the underlying causes [13, 14]. During the study period, computerized algorithms employing deterministic and probabilistic linkage were used to link multiple records pertaining to the same individual. In contrast to POGONIS, during the study period OCR employed a set of computerized rules to passively assign the site and histology of the primary malignancy. Given this passive process, OCR was not able to return to source documents for additional data or clarifications, nor was OCR able to incorporate from additional data sources such as POGONIS.

### Study population

All Ontario residents diagnosed with any malignancy between 2000 and 2011, less than 18 years of age at diagnosis, and treated and registered at a pediatric oncology center were identified using POGONIS and included. Patients with histiocytic disorders such as Langerhans cell histiocytosis or hemophagocytic lymphohistiocytosis were excluded given their variable inclusion in OCR over the study period. The study eligibility end date of 2011 was chosen to maximize the eligible cases be registered in both databases.

### Determining OCR diagnoses

As noted above, and in contrast to adult classification systems, pediatric cancers are generally categorized according to morphology and not primary site of origin. The third edition of the International Classification of Childhood Cancer (ICCC-3) is currently accepted as the standard classification system for childhood cancer [15]. The ICCC-3 operates hierarchically, with 12 main Level 1 diagnostic groups and 47 Level 2 diagnostic subgroups. For certain heterogeneous subgroups, Level 3 optional extended classifications are provided. As an example, Diagnostic Group I corresponds to leukemias, myeloproliferative diseases, and myeloplastic diseases, with Diagnostic Subgroup Ia pertaining to lymphoid leukemias and extended classification Ia.1 designating precursor cell leukemias. The ICCC-3 also includes an algorithm which converts International Classification of Diseases for Oncology, third edition (ICD-O-3), codes (ICD-O-M, ICD-O-T) to ICCC-3 diagnostic groups and subgroups [15]. ICCC-3 diagnostic groups are mainly based on morphology codes indicated by morphology (ICD-O-M), but are sometimes also dependent on topography codes (ICD-O-T) [16]. For example, cases with morphology codes indicating histologies consistent with germ cell tumors (e.g. 9071 – yolk sac tumor, 9080 – teratoma) are further classified by ICCC-3 as gonadal, intracranial/intraspinal, or extracranial/extragonadal based upon ICD-O-T codes.

**Fig. 1:**
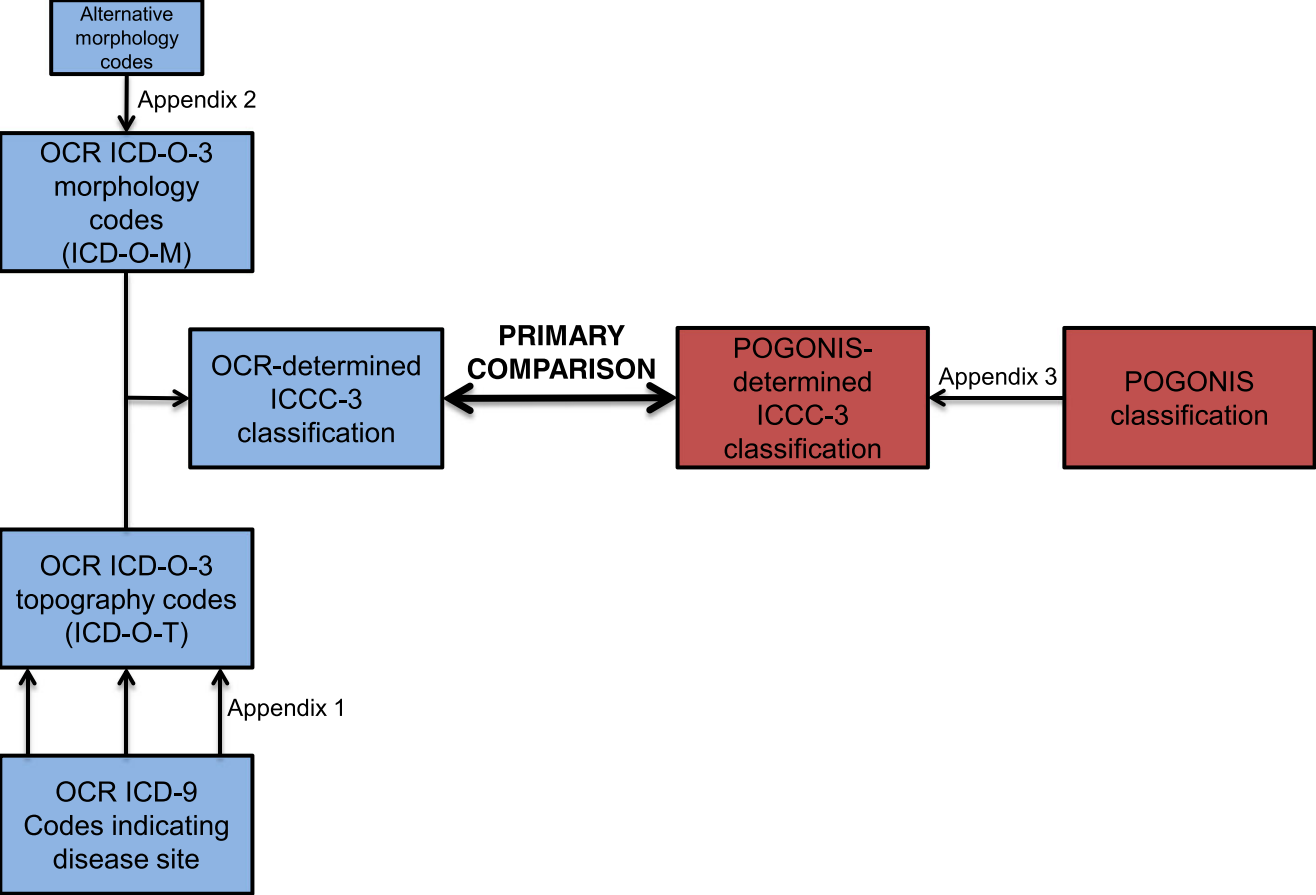
Schematic overview of study methodology and data sources

Similar to most population-based cancer registries, the OCR uses ICD-O-3 codes to classify incident cases. Using the aforementioned algorithm, we converted these ICD-O-3 codes to ICCC-3 diagnostic groups and subgroups (see Fig. 1 for schematic overview). As ICD-O-T codes were unavailable for the study population, we first converted OCR ICD-9 codes that indicated site of disease to ICD-O-T codes (Fig. 1). For example, we converted the ICD-9 code 189.0 (malignant neoplasm of kidney, except pelvis) and its derivatives to the ICD-O-T code C64.9, which indicates a primary renal site of disease. Additional examples may be seen in Additional file 1.

Importantly, as ICD-O-M codes were available from OCR, the information from these ICD-O-M codes were the only data source used to determine histology/morphology, even if additional or contrary information was available from the OCR ICD-9 codes. This decision was made as the published conversion algorithm to ICCC-3 uses only ICD-O-M codes for histology/morphology data and not ICD-9 codes [15]. Our approach should thus mimic that endorsed by the literature for use by researchers and cancer registrars.

Several additional modifications were necessary:

1. A small number of morphology codes from either previous ICD-O editions or more recently introduced were encountered in the OCR. These codes were first mapped to ICD-O-3 morphology/histology codes before the conversion to ICCC-3 (Additional file 2).
2. Patients for whom no ICD-O-M code was listed or for whom codes indicating solely “malignant primary” were classified as Diagnostic Subgroup XIIb, or “Other unspecified malignant tumors”. Patients coded as 9990/3, or “No mircoscopic proof”, were similarly classified. As noted above, ICD-9 codes were not used in these cases in order to clarify histology/morphology.
3. Rhabdoid tumors are rare malignancies that share a characteristic histology and which occur primarily in the brain (known as atypical teratoid/rhabdoid tumors – AT/RT) and kidney [17]. The ICCC-3 algorithm classifies any tumor with ICD-O-M code 9508/3 (AT/RT) as an AT/RT (IIIc.4) regardless of site, and tumors with ICD-O-M code 8963/3 (malignant rhabdoid tumor) as either rhabdoid renal tumors (VIa.2) or extrarenal rhabdoid tumors (IXd.3) depending on site. The algorithm however does not account for patients with ICD-O-M code 8963/3 with a central nervous system primary site. We classified these patients as having AT/RT (IIIc.4).

### Determining POGONIS diagnoses

POGONIS was established in 1985, prior to the existence of internationally recognized classification systems for childhood cancer. Malignancies are therefore categorized in POGONIS using a unique classification system derived by local clinicians that nonetheless shares similarities with the ICCC-3. Each POGONIS diagnosis code was mapped to the appropriate ICCC-3 category; an example is illustrated in Additional file 3. Rare pediatric malignancies such as squamous cell carcinomas and malignant carcinoid tumors were coded in POGONIS as single diagnostic categories independent of site, unlike in the ICCC-3. For these tumors, site of disease information was also extracted from POGONIS to allow accurate ICCC-3 categorization. Rare cases for which only general diagnoses were available (e.g. “bone tumor”) were mapped to ICCC-3 Level 1 diagnostic groups only. Other demographic variables were also obtained from POGONIS, including age at diagnosis, gender, and time period (early, 2000–2005 vs. late, 2006–2011).

### Validation of OCR diagnoses

Cohort patients were linked to the OCR deterministically by individually assigned Ontario Health Insurance Program numbers. Where deterministic linkage was not possible due to a lack of an exact health insurance number match, probabilistic linkage using name, date of birth and gender was employed. All patients linked probabilistically were reviewed for linkage quality. For those patients successfully linked, OCR-based and POGONIS-based ICCC-3 classifications were compared. Comparisons were made by Level 1 diagnostic groups and where appropriate, Level 2 and Level 3 subgroups. Given its use of pediatric-trained data managers, active capture of data, clinician involvement, and ability to correct and supplement data when needed, the POGONIS-based classification was considered the gold standard against which the OCR-based classification was validated.

### Analyses

Successfully and unsuccessfully linked patients were compared by age, gender, and time period of diagnosis using the Chi square test or the Wilcoxon rank sum test as appropriate. Guidelines pertaining to studies validating health administrative data have recommended the use of multiple statistical measures of agreement [18]. Agreement between the POGONIS-based and OCR-based classifications was therefore assessed by calculating the kappa statistic, sensitivity, specificity, positive predictive value and negative predictive value. Statistical significance was defined as *p* < 0.05.

## Results

**Fig. 2:**
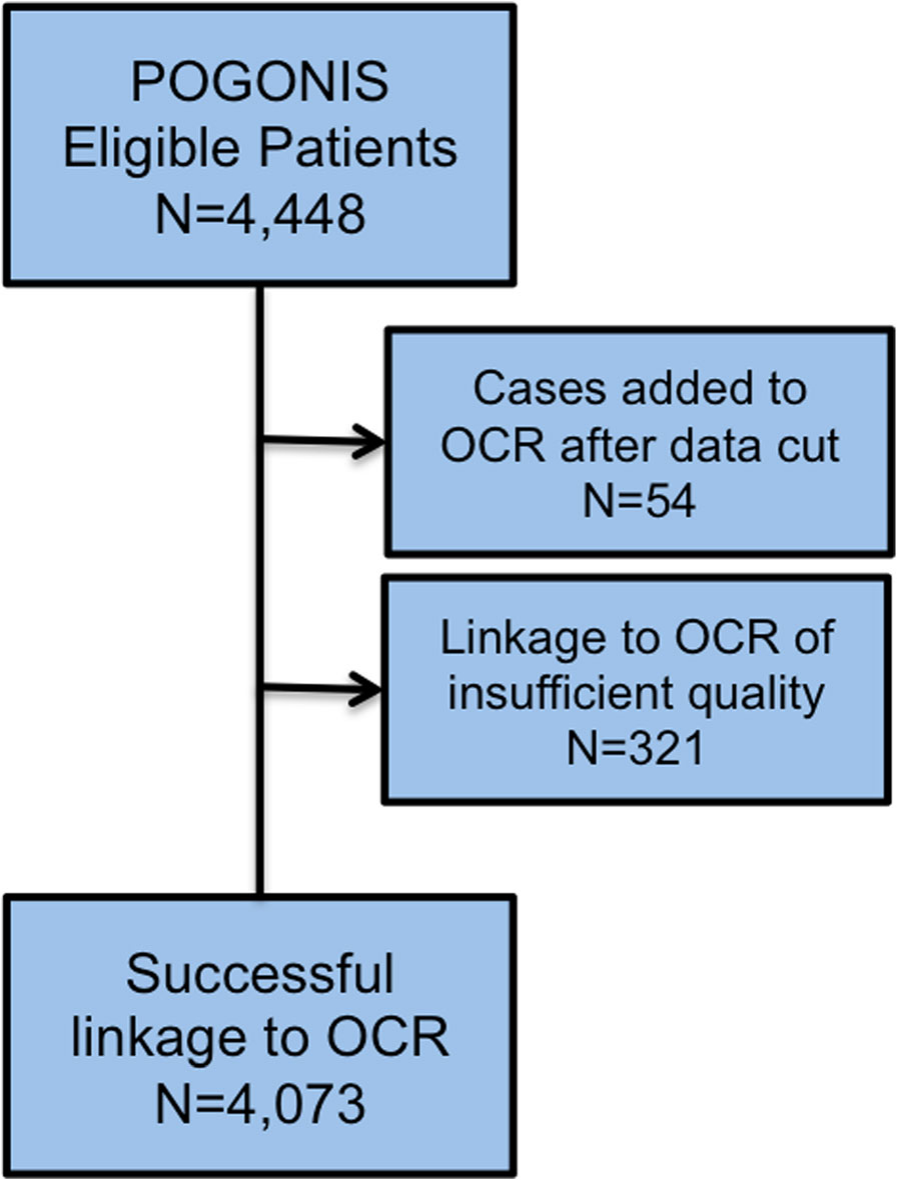
Patient linkage

Using POGONIS, 4448 patients were identified as meeting study cohort inclusion criteria (Fig. 2). Of these, 4073 (91.6 %) were successfully linked to the OCR. Linked and unlinked patients showed no differences in age [median 6 years, (interquartile range - IQR 3–13) vs. 8 years (2–13); *p* = 0.53] or gender [2223/4073 (54.6 %) male vs. 196/374 (52.4 %); *p* = 0.42]. Unlinked patients were more likely to have been diagnosed in the late time period [170/375 (45.3 %) vs. 1598/4073 (35.9 %); *p* = 0.02]. Of the 375 unlinked patients, 54 were subsequently added to the OCR after the data cut, accounting for the time period findings. The remaining 321 unlinked cases were deemed to have linkages of insufficient quality. Of the 4073 successfully linked patients, 3693 (90.7 %) were linked deterministically by health card number. A total of 380 (9.3 %) were linked probabilistically, with 357 (93.9 %) linked based on exact date of birth and a phonetic encoding of surname and the remaining based on various combinations of the linkage variables.

Mapping POGONIS diagnostic codes to ICCC-3 resulted in the successful assignment of ICCC-3 Level 1 diagnostic groups to all patients and of Level 2 diagnostic subgroups to all but 25 (0.6 %) patients. These latter 25 were retained in the linkage exercise. Mapping OCR codes resulted in the successful assignment of ICCC-3 diagnostic groups and subgroups to all patients. Table 1 illustrates the number of children classified by POGONIS and by the OCR into each ICCC-3 Level 1 diagnostic group, as well as the sensitivity, specificity, and positive and negative predictive values of the OCR-based diagnostic categories as compared to the gold standard POGONIS-based categories. The kappa statistics of agreement are also listed. With the exception of Diagnostic Group XII (Other and unspecified malignant neoplasms), kappas were generally excellent (>0.80), [19] ranging from 0.86 to 1.00. Again excepting Diagnostic Group XII, sensitivities ranged from 0.82 to 1.00. While POGONIS classified only 23 (0.5 %) patients in diagnostic group XII, using OCR data resulted in 259 (6.3 %) patients classified thusly.

**Table 1:** Measures of agreement between OCR-based and POGONIS-based diagnostic groups

Malignancy type	N, POGONIS	N, OCR	Kappa (95 % CI)	Sensitivity	Specificity	PPV	NPV
I. Leukemias, myeloproliferative diseases, and myelodysplastic diseases	1305	1143	0.89 (0.88–0.91)	0.87	1.00	0.99	0.94
II. Lymphomas and reticuloendothelial neoplasms	585	554	0.90 (0.88–0.92)	0.89	0.99	0.94	0.98
III. CNS and miscellaneous intracranial and intraspinal neoplasms	790	757	0.95 (0.94–0.96)	0.94	1.00	0.98	0.99
IV. Neuroblastoma and other peripheral nervous cell tumors	262	256	0.97 (0.96–0.99)	0.96	1.00	0.98	1.00
V. Retinoblastoma	105	105	1.00 (1.00–1.00)	1.00	1.00	1.00	1.00
VI. Renal tumors	174	175	0.98 (0.96–0.99)	0.98	1.00	0.98	1.00
VII. Hepatic tumors	68	64	0.92 (0.88–0.97)	0.90	1.00	0.95	1.00
VIII. Malignant bone tumors	181	192	0.89 (0.86–0.92)	0.92	0.99	0.87	1.00
IX. Soft tissue and other extraosseus sarcomas	285	260	0.86 (0.82–0.89)	0.82	0.99	0.91	0.99
X. Germ cell tumors, trophoblastic tumors, and neoplasms of gonads	140	136	0.96 (0.93–0.98)	0.94	1.00	0.97	1.00
XI. Other malignant epithelial neoplasms and malignant melanomas	155	172	0.93 (0.90–0.96)	0.98	0.99	0.88	1.00
XII. Other and unspecified malignant neoplasms	23	259	0.06 (0.02–0.10)	0.43	0.94	0.04	1.00

*CI* confidence interval, *CNS* central nervous system, *N* number, *NPV* negative predictive value, *OCR* Ontario Cancer Registry, *POGONIS* Pediatric Oncology Group of Ontario Networked Information System, *PPV* positive predictive value

Table 2 illustrates the same parameters for selected ICCC-3 diagnostic subgroups (Level 2 and 3). Measures of agreement remained excellent for some subgroups, including Hodgkin lymphoma, neuroblastoma, nephroblastoma, hepatoblastoma and osteosarcoma. However, agreement for several diagnostic subgroups was significantly inferior, with kappa statistics of 0.77 for acute lymphoblastic leukemia (ALL), 0.71 for non-rhabdomyosarcoma soft tissue sarcomas, 0.24 for lymphoblastic lymphomas, and 0.02 for Burkitt lymphomas.

**Table 2:** Measures of agreement between selected OCR-based and POGONIS-based diagnostic subgroups

Malignancy type	N, POGONIS	N, OCR	Kappa (95 % CI)	Sensitivity	Specificity	PPV	NPV
Ia.1. Precursor cell leukemias (acute lymphoblastic leukemia)	1070	775	0.77 (0.75–0.80)	0.71	0.99	0.98	0.91
Ib. Acute myeloid leukemias	187	164	0.84 (0.81–0.89)	0.80	1.00	0.91	0.99
IIa. Hodgkin lymphomas	312	310	0.99 (0.98–1.00)	0.98	1.00	0.99	1.00
IIb-IId, IIe. Non Hodgkin lymphomas	271	244	0.78 (0.74–0.82)	0.76	0.99	0.84	0.98
IIb.1 Precursor cell lymphomas (lymphoblastic lymphomas)	67	22	0.24 (0.12–0.36)	0.16	1.00	0.50	0.99
IIc. Burkitt lymphomas	78	1	0.02 (0.02–0.07)	0.01	1.00	1.00	0.98
IIIb. Astrocytomas	446	343	0.82 (0.79–0.85)	0.74	1.00	0.96	0.97
IIIa1. Ependymomas	72	69	0.95 (0.91–0.99)	0.93	1.00	0.97	1.00
IIIc. Intracranial and intraspinal embryonal tumors	199	201	0.91 (0.87–0.94)	0.91	1.00	0.91	1.00
IIIc.1 Medulloblastomas	152	134	0.91 (0.88–0.95)	0.86	1.00	0.98	1.00
IIIx. All other CNS tumors	73	144	0.34 (0.26–0.43)	0.53	0.97	0.27	0.99
IVa. Neuroblastoma and ganglioneurblastoma	259	254	0.97 (0.96–0.99)	0.97	1.00	0.98	1.00
VIa.1 Nephroblastoma	141	140	0.98 (0.97–1.00)	0.98	1.00	0.99	1.00
VIIa. Hepatoblastoma	58	56	0.96 (0.93–1.00)	0.95	1.00	0.98	1.00
VIIIa. Osteosarcoma	88	87	0.96 (0.93–0.99)	0.95	1.00	0.97	1.00
VIIIc. Ewing tumor and related sarcomas of bone	82	93	0.81 (0.74–0.87)	0.87	0.99	0.76	1.00
IXa. Rhabdomyosarcomas	116	128	0.92 (0.89–0.96)	0.97	1.00	0.88	1.00
IXb-IXe. Non-rhabdomyosarcoma soft tissue sarcomas	153	132	0.71 (0.65–0.77)	0.67	0.99	0.78	0.99

*CI* confidence interval, *CNS* central nervous system, *N* number, *NPV* negative predictive value, *OCR* Ontario Cancer Registry, *POGONIS* Pediatric Oncology Group of Ontario Networked Information System, *PPV* positive predictive value

We then more closely examined reasons for discrepancy among the above three diagnostic subgroups (ALL, lymphoblastic lymphoma, Burkitt lymphoma), representing common childhood cancers but with poor measures of agreement. Of 1070 patients classified by POGONIS as having ALL, 311 (29.1 %) were improperly classified by the OCR. The most common OCR ICD-O-M codes found among these children were 9990/3 (*N* = 119, 38.2 % - clinically malignant tumor), 9831/3 (*N* = 118, 37.9 % - chronic lymphoproliferative disorder of NK-cells) and 9801/3 (*N* = 23, 7.4 % - acute leukemia, NOS). Interestingly, 244/311 (78.5 %) were correctly identified as having ALL by OCR ICD-9 codes. 16 children were incorrectly diagnosed as having ALL by OCR; the most common correct diagnoses in POGONIS for these children were acute mixed-lineage leukemia (*N* = 6, 37.5 %) and lymphoblastic lymphoma (*N* = 5, 31.3 %).

Of 67 children with lymphoblastic lymphoma according to POGONIS data, 56 (83.6 %) were misclassified by the OCR. The most common OCR ICD-O-M code found among these patients was 9590/3 (37, 66.1 % - malignant lymphoma, NOS). Using OCR ICD-9 codes would not have correctly identified these children; 36 (64.3 %) were coded as having “other lymphomas” while another 10 (17.9 %) were coded as having “lymphosarcoma”. 11 children were incorrectly identified as having lymphoblastic lymphoma by OCR; according to POGONIS, 8 (72.7 %) of these children in fact had ALL.

Of the 78 children categorized as having Burkitt lymphoma by POGONIS, nearly all were misclassified by the OCR (77, 98.7 %). The most common OCR ICD-O-M code found among these patients was 9750/3 (68, 88.3 % - malignant histiocytosis). Using OCR ICD-9 codes would have correctly identified 68 (88.3 %) of these patients.

## Discussion

To our knowledge, this is the first study to systematically examine the validity of diagnosis data for cases of childhood cancer in a general cancer registry. In doing so, we have shown that while excellent agreement was achieved within most broad diagnostic categories and for some specific diagnoses, agreement among several common childhood malignancies was poor to dismal.

The predominant source of error was the derivation of incorrect ICD-O-M codes from source documents, and the consequent assignment of inappropriate ICCC-3 diagnostic groups and/or subgroups. This was in most cases due to the inappropriate assignment of patients with specific diagnoses to “other” or “miscellaneous” categories, as reflected by the higher number of patients in such categories based on OCR data as compared to POGONIS data, as opposed to misclassification among different specific types of childhood cancer. This was apparent both within specific diagnostic groups (e.g. 73 POGONIS-assigned patients to the “other central nervous system - CNS - tumors” subgroup as compared to 144 OCR-assigned patients) and overall (23 versus 259 patients in Diagnostic Group XII – other and unspecified malignant neoplasms).

The magnitude of this problem varied between diagnostic subgroups. The OCR was able to consistently identify unique childhood solid tumors (e.g. neuroblastoma, retinoblastoma, Wilms tumor, hepatoblastoma) as demonstrated by near perfect, and in some cases perfect, measures of agreement. Hematologic and CNS cancers were however more problematic. For example, a diagnosis of ALL, the most common childhood cancer, in OCR carried a sensitivity of only 0.71. Almost no patient with Burkitts lymphoma was correctly identified by the OCR. Previous studies in adults have identified similar issues among non-Hodgkin lymphomas (NHL). Clarke et al. examined the validity of NHL diagnoses among adults in the Greater Bay Area Cancer Registry, and found that agreement on subtype classification was only 59 %, with the positive predictive values of specific subtypes reaching as low as 0.05 [20]. In later work, the same group found that almost half of the adult lymphomas recorded as unclassifiable in their registry could in fact be assigned a specific subtype [21].

Our findings have important implications for individuals using cancer registry data. Population-based incidence derived from cancer registries for hematologic and CNS pediatric malignancies may represent significant underestimates, as a proportion of children with these cancers are misclassified into “miscellaneous” categories. More generally, this study demonstrates the pitfalls in designating passive cancer registry data as a gold standard in less common malignancies such as childhood cancer. Our results also illustrate the importance of conducting registry validation studies, particularly given their growing importance in guiding cancer policy [1].

Our results also indicate that pediatric cancer specific algorithms, training and personnel may be necessary to improve the accuracy of data pertaining to several common childhood malignancies. This may be particularly relevant to more complex and rare childhood cases. It is noteworthy that in several cases, the ICD-O-M was incorrectly assigned in OCR despite OCR ICD-9 codes that agreed with the POGONIS diagnosis. For example, nearly all patients diagnosed with Burkitts lymphoma according to POGONIS received ICD-O-M codes in OCR indicating “malignant histiocytosis”, despite OCR ICD-9 codes that in the majority of cases also indicated Burkitts lymphoma. This suggests that the accuracy of cancer registry morphology data, particularly in hematologic malignancies, may be improved, but not perfected by comparing it to ICD-9 data. Similarly, researchers wishing to identify cohorts of childhood cancer patients from registry data should consider validating algorithms based on ICD-O-3 codes, as suggested by the ICCC-3, against more complex algorithms that also incorporate ICD-9 codes prior to analyzing these cohorts. Finally, our findings also suggest that registries may benefit from automatically reviewing all pediatric cases assigned to “miscellaneous” or “unclassifiable” categories. Given the small number of pediatric cases relative to the overall cancer burden, implementing these strategies may not require significant additional resources. Their validity however remains theoretical and warrants further study. Indeed, the involvement of national and international bodies such as the International Agency for Cancer Research (IARC) and the International Association of Cancer Registries may aid in designing, implementing and evaluating these strategies.

One limitation of this study pertains to the generalizability of its results to other cancer registries. As noted above, during the study period the OCR relied upon passive algorithms to reconcile information on histology from several sources. Other cancer registries utilize other strategies; for example, the use of hospital-based computerized reporting systems that enable centers to transmit registry-reportable information [20]. Whether the validity of diagnostic data collected by registries using these alternative strategies differs from that in the current study is unknown, though the study cited above demonstrating the poor validity of NHL diagnoses in adults was conducted using such a cancer registry. Interestingly however, in a report from the Surveillance, Epidemiology, and End Results (SEER) group of cancer registries on childhood cancer between 1975–1995, only 0.3–0.8 % of cases were classified in Diagnostic Group XII (Other and unspecified malignant neoplasms), [22] in line with the 0.5 % in POGONIS in the current study but below the 6.3 % of cases in the OCR. The OCR is in fact currently developing new algorithms and allowing for manual edits where applicable; future work will examine the impact of these changes on data validity.

Strengths of this study include its population-based nature and large sample size, allowing conclusions to be drawn for specific diagnostic subgroups. This was feasible due to the presence of an independent active childhood cancer registry. In many jurisdictions where no such childhood cancer registry exists, validation would require more onerous chart abstraction. Several additional limitations however also merit notice. First, we were unable to link 9.4 % of cohort patients to OCR data. We cannot rule out systematic differences between these patients and the rest of the cohort that may have impacted our results. Second, our study did not have access to ICD-O-T codes, though ICD-9 codes indicating site were used as a substitute. Third, the premise of the POGONIS diagnosis as gold standard is an assumption; this assumption is however reasonable given active and near real-time data entry with clinician oversight. It should also be noted however that while POGONIS may be considered complete and population-based for children aged <15 years at diagnosis, it is not for those aged 15 or older. OCR by contrast is of course population-based for all ages. Indeed the completeness of OCR has been demonstrated in a prior comparison with POGONIS data [11].

## Conclusions

In conclusion, we have shown that while the validity of OCR data was generally excellent across most broad diagnostic groups and some specific subgroups, misclassification of hematologic and CNS tumors among children was common. These findings have important implications for those using cancer registry data to conduct pediatric cancer research or guide policy. Though more active methods of cancer registration may theoretically result in greater validity, validation studies of pediatric data in other cancer registries should be conducted to identify practices and procedures leading to the highest quality information.

## Additional files

Additional file 1: Examples of converting ICD-9 codes indicating disease site to ICD-O-T codes. (DOCX 15 kb)
Additional file 2: Mapping of ICD-O morphology codes used by the Ontario Cancer Registry but not accounted for by the ICCC-3 algorithm. (DOCX 14 kb)
Additional file 3: Mapping of POGONIS diagnostic groups pertaining to central nervous tumors of neuroepithelial tissue to ICCC-3 diagnostic groups and subgroups. (DOCX 106 kb)

## Abbreviations

AT/RT: Atypical teratoid/rhabdoid tumor
CI: Confidence interval
CNS: Central nervous system
ICCC-3: International classification of childhood cancer, 3^rd^ edition
ICD-O-3: International classification of disease for oncology, 3^rd^ edition
ICD-O-M: International classification of diseases, morphology
ICD-O-T: International classification of diseases, topography
OCR: Ontario cancer registry
POGONIS: Pediatric oncology group of Ontario networked information system.
