# Supplementary material for: The validity of pediatric cancer diagnoses in a population-based general cancer registry in Ontario, Canada
Source: BMC Cancer. 2016 Nov 14;16:885. doi: 10.1186/s12885-016-2931-8 (PMC5109739; doi:10.1186/s12885-016-2931-8)
Supplement: Additional file 1: — Examples of converting ICD-9 codes indicating disease site to ICD-O-T codes. (DOCX 15 kb) [file 12885_2016_2931_MOESM1_ESM.docx]

Additional file 1. Examples of converting ICD-9 codes indicating disease site to ICD-O-T codes

| ICD-9 | | ICD-O-T | |
| --- | --- | --- | --- |
| 147 | Malignant neoplasm of nasopharynx | C11.0-C11.9 | Nasopharynx |
| 155 | Malignant neoplasm of liver and intrahepatic bile ducts | C22.0-C22.1 | Liver and intrahepatic bile ducts |
| 170 | Malignant neoplasm of bone and articular cartilage | C40.0-C40.9 | Bones, joints and articular cartilage of limbs |
|  |  | C41.0-C41.9 | Bones, joints and articular cartilage of other and unspecified sites |
| 183 | Malignant neoplasm of ovary and other uterine adnexa | C56.9 | Ovary |
| 186 | Malignant neoplasm of testis | C62.0-C62.9 | Testis |
| 189.0 | Malignant neoplasm of kidney, except pelvis | C64.9 | Kidney |
| 191 | Malignant neoplasm of brain | C71.0-C71.9 | Brain |
| 192.0 | Malignant neoplasm of cranial nerve | C72.3 | Optic nerve |
| 193 | Malignant neoplasm of thyroid gland | C73.9 | Thyroid gland |
| 194.0 | Malignant neoplasm of adrenal gland | C74.0-C74.0 | Adrenal gland |
| 194.3 | Malignant neoplasm of pituitary gland | C75.1 | Pituitary gland |
