# Supplementary material for: The validity of pediatric cancer diagnoses in a population-based general cancer registry in Ontario, Canada
Source: BMC Cancer. 2016 Nov 14;16:885. doi: 10.1186/s12885-016-2931-8 (PMC5109739; doi:10.1186/s12885-016-2931-8)
Supplement: Additional file 2: — Mapping of ICD-O morphology codes used by the Ontario Cancer Registry but not accounted for by the ICCC-3 algorithm. (DOCX 14 kb) [file 12885_2016_2931_MOESM2_ESM.docx]

Additional file 2. Mapping of ICD-O morphology codes used by the Ontario Cancer Registry but not accounted for by the ICCC-3 algorithm

| Morphology Code (ICD-O Version) | N | Description | Equivalent ICD-O-3 Morphology Code in ICCC-3 algorithm | ICCC-3 Subgroup Description |
| --- | --- | --- | --- | --- |
| 9656 (ICD-O-1) | 242 | Nodular sclerosis classical Hodgkin lymphoma | 9663 | Hodgkin lymphoma, nodular sclerosis, NOS |
| 9657 (ICD-O-2) | 5 | Hodgkin’s disease, lymphocytic predominance, NOS | 9651 | Hodgkin lymphoma, lymphocyte-rich |
| 9811 (ICD-O-3, effective 2010) | 2 | B lymphoblastic leukemia/lymphoma, NOS | 9835 | Precursor cell lymphoblastic leukemia, NOS |
| 9821 (ICD-O-2) | 766 | Acute lymphoblastic leukemia, L1 | 9835 | Precursor cell lymphoblastic leukemia, NOS |
| 9971 (ICD-O-3, effective 2010) | 1 | Polymorphous PTLD | 9970 | Lymphoproliferative disorder, NOS |
