# Supplementary material for: The validity of pediatric cancer diagnoses in a population-based general cancer registry in Ontario, Canada
Source: BMC Cancer. 2016 Nov 14;16:885. doi: 10.1186/s12885-016-2931-8 (PMC5109739; doi:10.1186/s12885-016-2931-8)
Supplement: Additional file 3: — Mapping of POGONIS diagnostic groups pertaining to central nervous tumors of neuroepithelial tissue to ICCC-3 diagnostic groups and subgroups. (DOCX 106 kb) [file 12885_2016_2931_MOESM3_ESM.docx]

Additional File 3. Mapping of POGONIS diagnostic groups pertaining to central nervous tumors of neuroepithelial tissue to ICCC-3 diagnostic groups and subgroups

| POGONIS Diagnostic Group | ICCC-3 Subgroup | ICCC-3 Subgroup Description |
| --- | --- | --- |
| Glial tumors |  |  |
| Astrocytic tumors |  |  |
| Astrocytoma, optic tract | IIIb | Astrocytomas |
| Astrocytoma, brainstem | IIIb | Astrocytomas |
| Astrocytoma, posterior fossa | IIIb | Astrocytomas |
| Astrocytoma, spinal cord | IIIb | Astrocytomas |
| Astrocytoma, other sites | IIIb | Astrocytomas |
| Anaplastic astrocytoma | IIIb | Astrocytomas |
| Subependymal giant cell tumors | IIIb | Astrocytomas |
| Gigantocellular glioma | IIIb | Astrocytomas |
| Oligodendroglial tumours |  |  |
| Oligodendroglioma | IIId.1 | Oligodendrogliomas |
| Anaplastic oligodendroglioma | IIId.1 | Oligodendrogliomas |
| Ependymal tumors |  |  |
| Ependymoma | IIIa.1 | Ependymomas |
| Anaplastic ependymoma | IIIa.1 | Ependymomas |
| Myxopapillary ependymoma | IIIa.1 | Ependymomas |
| Choroid plexus tumors |  |  |
| Choroid plexus papilloma | IIIa.2 | Choroid plexus tumor |
| Anaplastic choroid plexus tumours/carcinoma | IIIa.2 | Choroid plexus tumor |
| Mixed glioma (low grade) |  |  |
| Anaplastic mixed glioma | IIId.2 | Mixed and unspecified gliomas |
| Glioblastomatous tumors |  |  |
| Glioblastoma multiforme | IIIb | Astrocytomas |
| Others | IIIb | Astrocytomas |
| Gliomatosis cerebri | IIId.3 | Neuroepithelial glial tumors of uncertain origin |
| Optic pathway glioma, not biopsied | IIIb | Astrocytomas |
| Brainstem glioma, not biopsied | IIIb | Astrocytomas |
| Neuronal tumors |  |  |
| Gangliocytoma | IIIe.4 | Neuronal and mixed neuronal-glial tumors |
| Ganglioglioma | IIIe.4 | Neuronal and mixed neuronal-glial tumors |
| Anaplastic ganglioglioma | IIIe.4 | Neuronal and mixed neuronal-glial tumors |
| “Primitive” neuroepithelial tumors (PNET) |  |  |
| Medulloblastoma | IIIc.1 | Medulloblastomas |
| PNET with other elements | IIIc.2 | PNET |
| Medulloepithelioma | IIIc.3 | Medulloepithelioma |
| Supratentorial PNET | IIIc.2 | PNET |
| Pineal cell tumors |  |  |
| Pineoblastoma | IIIe.3 | Pineal parenchymal tumors |
| Pineocytoma | IIIe.3 | Pineal parenchymal tumors |
